# Supplementary material for: Hepatocyte Proteome Alterations Induced by Individual and Combinations of Common Free Fatty Acids
Source: Int J Mol Sci. 2022 Mar 20;23(6):3356. doi: 10.3390/ijms23063356 (PMC8951603; doi:10.3390/ijms23063356)
Supplement: Supplementary file 1 [file ijms-23-03356-s001.zip › ijms-1623971-supplementary.pdf]

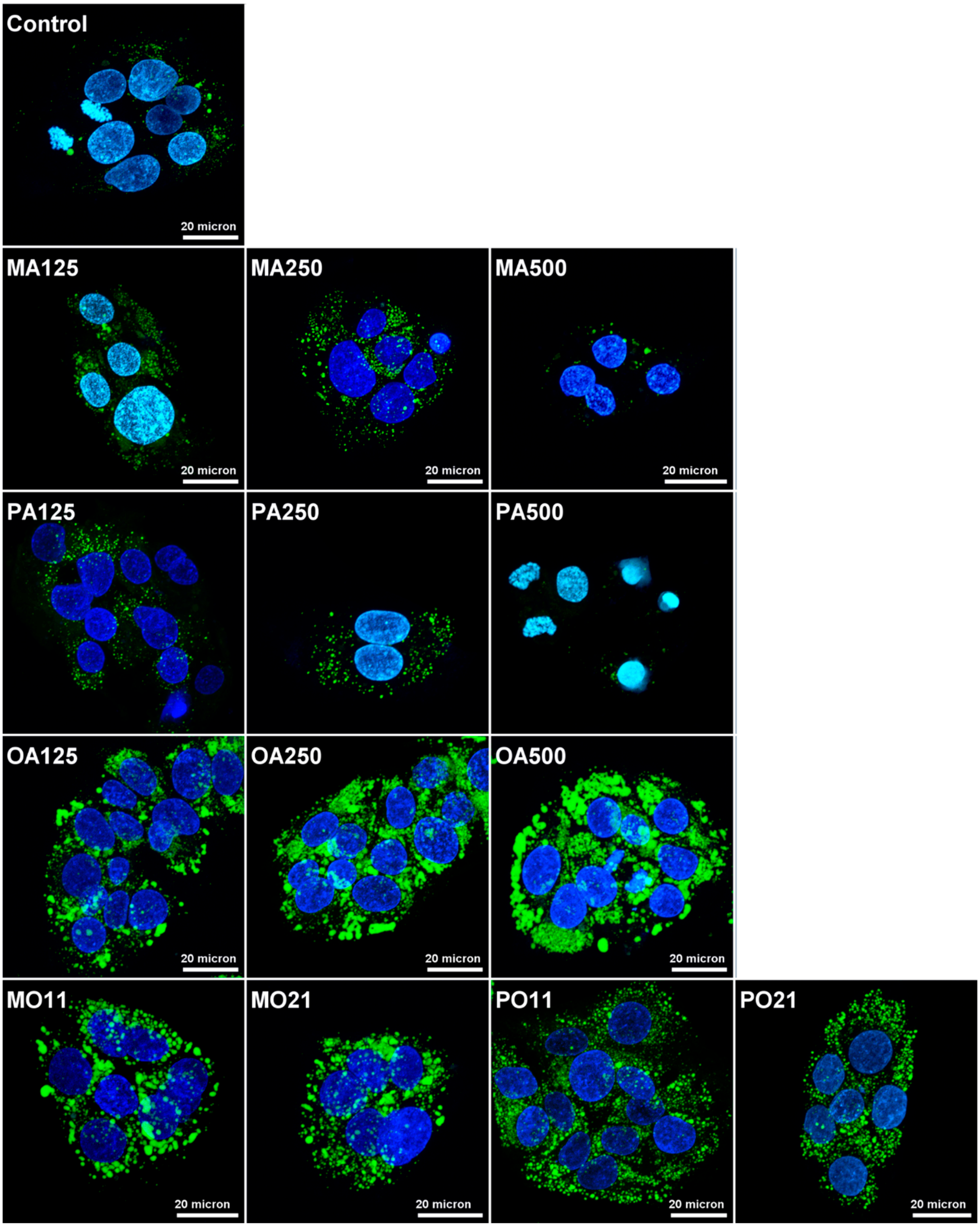

Figure S1: Representative images of HepG2 cells after lipid loading for 24 hours. LDs stained with BODIPY^{TM} 493/503 and nuclei with DAPI (LD= green; nuclei= blue). LD: lipid droplets; MA: myristic acid; PA: palmitic acid; OA: oleic acid; MO: myristic: oleic acid; PO: palmitic: oleic acid; 11: 125µM: 125 µM; 21: 250 µM: 125 µM.

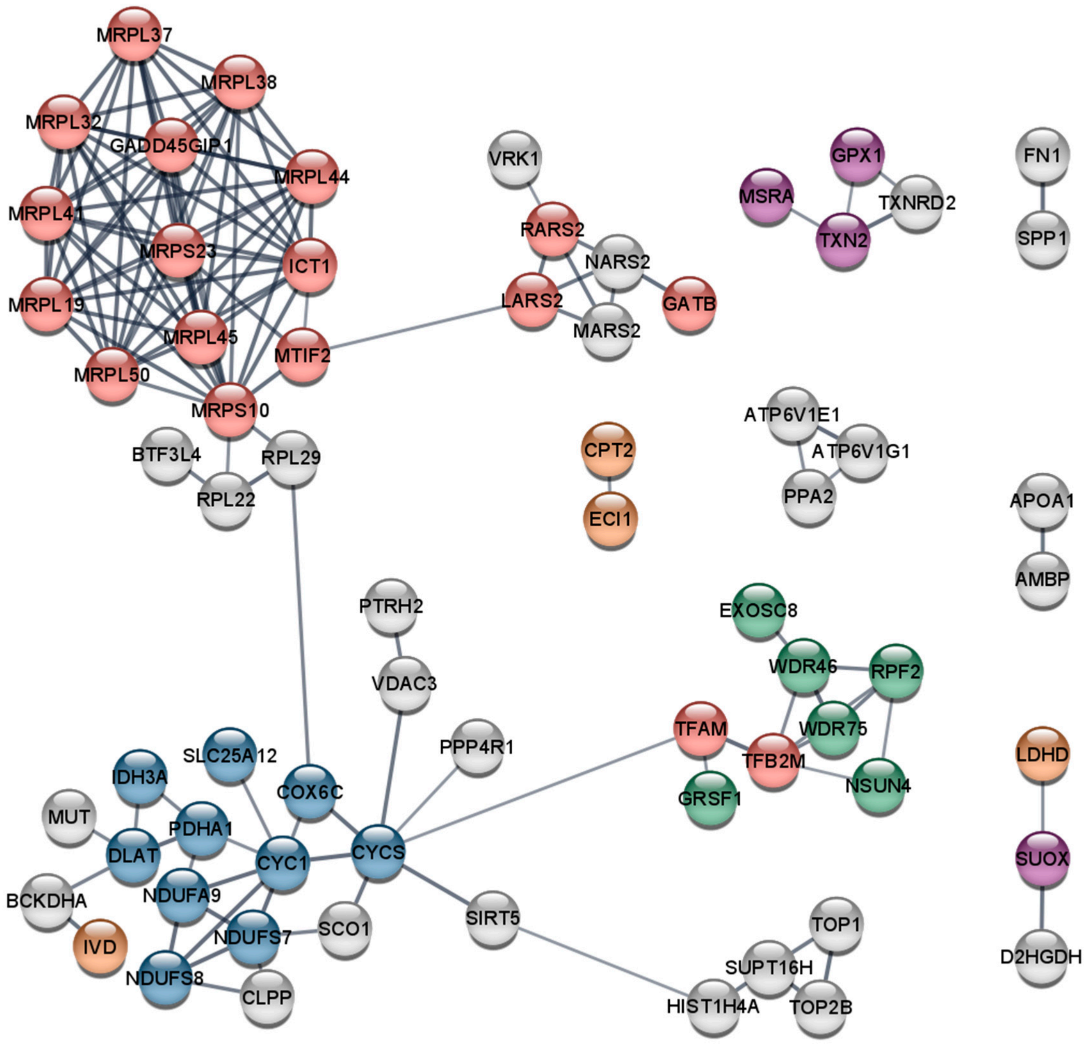

GO-term	strength	FDR
Mitochondrial gene expression	1.33	4.94e-15
Cellular respiration	1.05	4.59e-06
Monocarboxylic acid catabolic process	0.91	0.0317
ncRNA metabolic process	0.69	0.00058
Sulfur compound metabolic process	0.66	0.0237

Figure S2: String analysis of proteins exhibiting a ≥1.5-fold change following MA500 treatment. The minimum required interaction score was set to 0.7 (high confidence) and disconnected nodes were excluded from the display. Nodes were coloured correspondingly to selected GO biological process search results. MA treatment resulted in an upregulation of proteins associated with mitochondrial protein synthesis as well as oxidative phosphorylation. MA500: 500 µM myristic acid for 24 h.

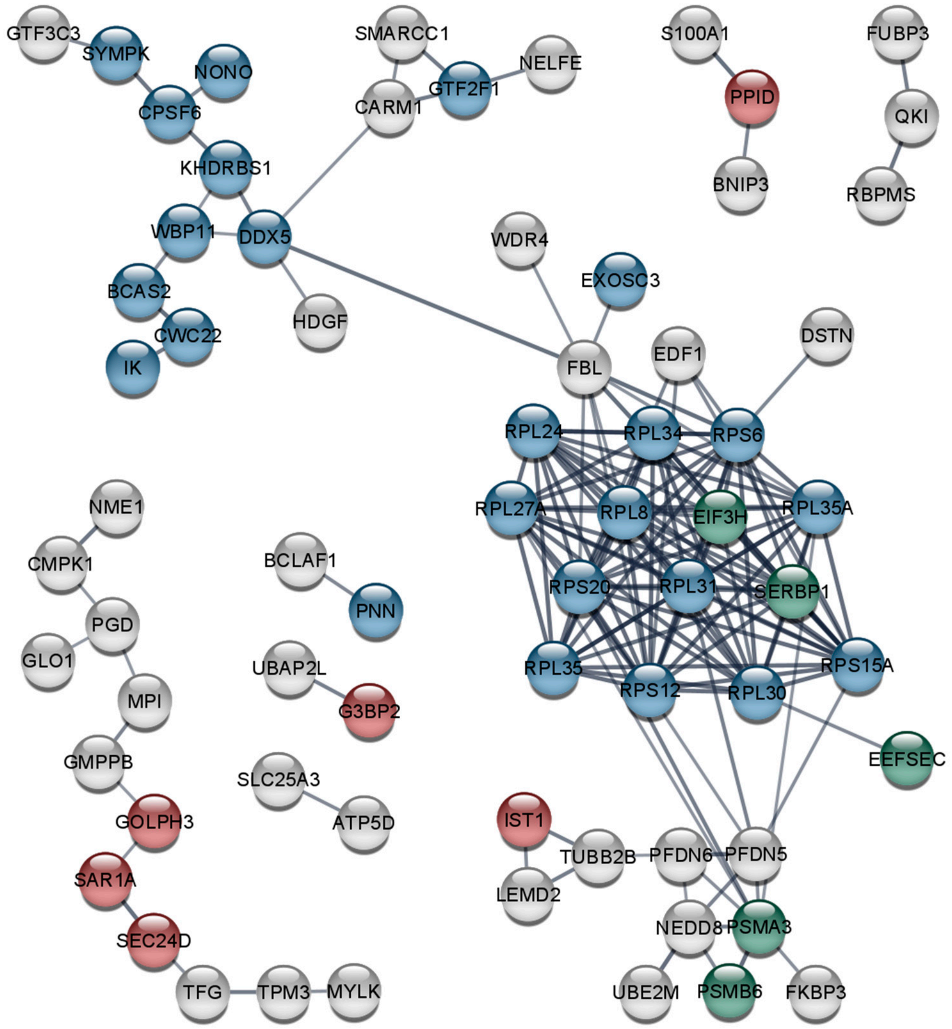

GO-term	strength	FDR
mRNA metabolic process	0.73	2.34e-08
Nitrogen compound transport	0.35	0.0054
Posttranscriptional regulation of gene expression	0.0059	4.94e-15

Figure S3: String analysis of proteins exhibiting a ≥-1.5-fold change following MA500 treatment. The minimum required interaction score was set to 0.7 (high confidence) and disconnected nodes were excluded from the display. Nodes were coloured corresponding to selected GO biological process search. MA treatment resulted in a downregulation of ribosomal proteins indicating a reduction in cellular growth. MA500: 500 µM myristic acid for 24 h.

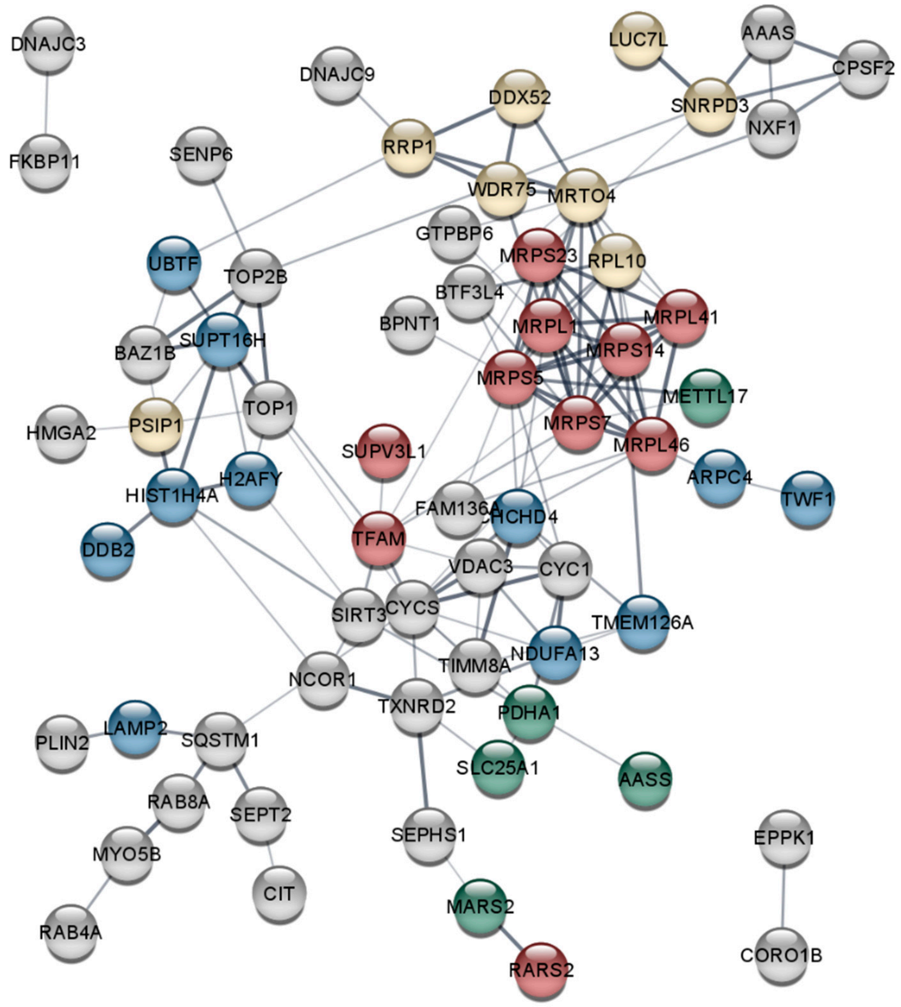

GO-term	strength	FDR
Mitochondrial gene expression	1.22	1.41e-05
Ribonucleoprotein complex biogenesis	0.75	0.0062
Cellular amide metabolic process	0.67	0.00073
Protein-containing complex subunit organization	0.59	2.14e-05

Figure S4: String analysis of proteins exhibiting a ≥1.5-fold change following PA500 treatment. The minimum required interaction score was set to 0.7 (high confidence) and disconnected nodes were excluded from the display. Nodes were coloured correspondingly to selected GO biological process search results. PA treatment resulted in an upregulation of proteins associated with mitochondrial protein synthesis.PA500: 500 µM palmitic acid for 24 h.

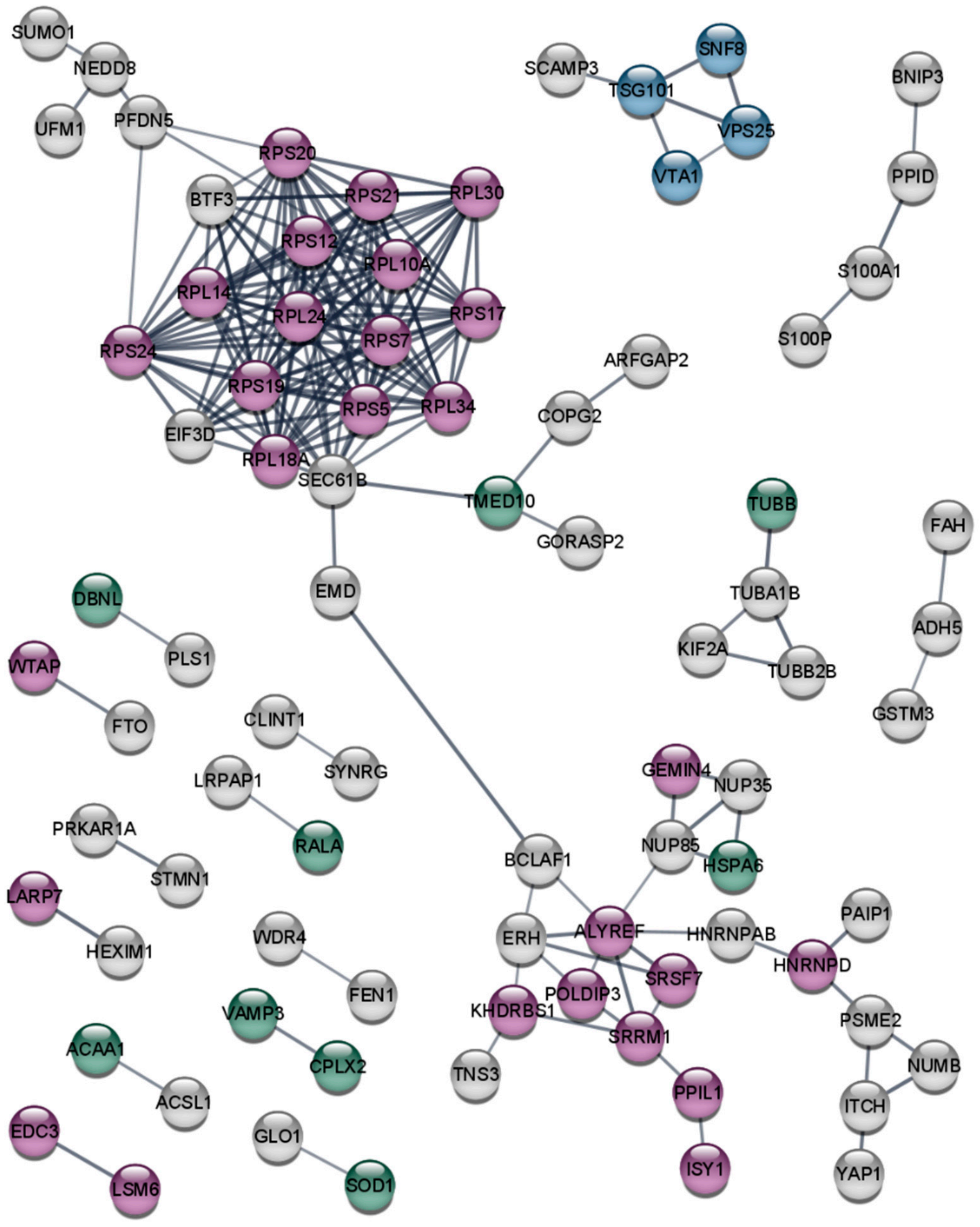

GO-term	strenght	FDR
Multivesicular body assembly	1.21	0.0257
mRNA metabolic process	0.8	2.94e-13
Exocytosis	0.46	0.0123

Figure S5: String analysis of proteins exhibiting a ≥-1.5-fold change following PA500 treatment. The minimum required interaction score was set to 0.7 (high confidence) and disconnected nodes were excluded from the display. Nodes were coloured correspondingly to selected GO biological process search. PA treatment resulted in a downregulation of ribosomal proteins indicating a reduction of cellular growth. PA500: 500 µM palmitic acid for 24 h.

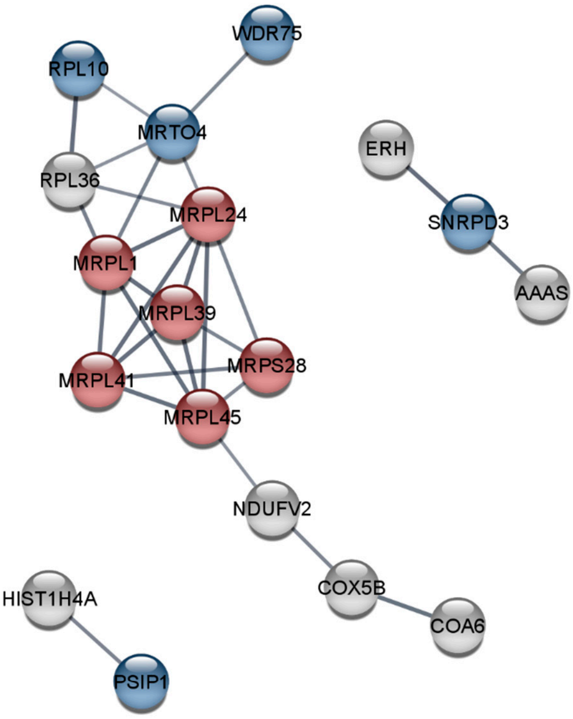

GO-term	strength	FDR
Mitochondrial gene expression	1.3	5.34e-05
Ribonucleoprotein complex biogenesis	0.83	0.0124

Figure S6: String analysis of proteins exhibiting a ≥1.5-fold change following OA500 treatment. The minimum required interaction score was set to 0.7 (high confidence) and disconnected nodes were excluded from the display. Nodes were coloured correspondingly to selected GO biological process search results. Treatment with OA led to an increase of proteins associated with mitochondrial protein synthesis. OA500: 500 µM oleic acid for 24 h.

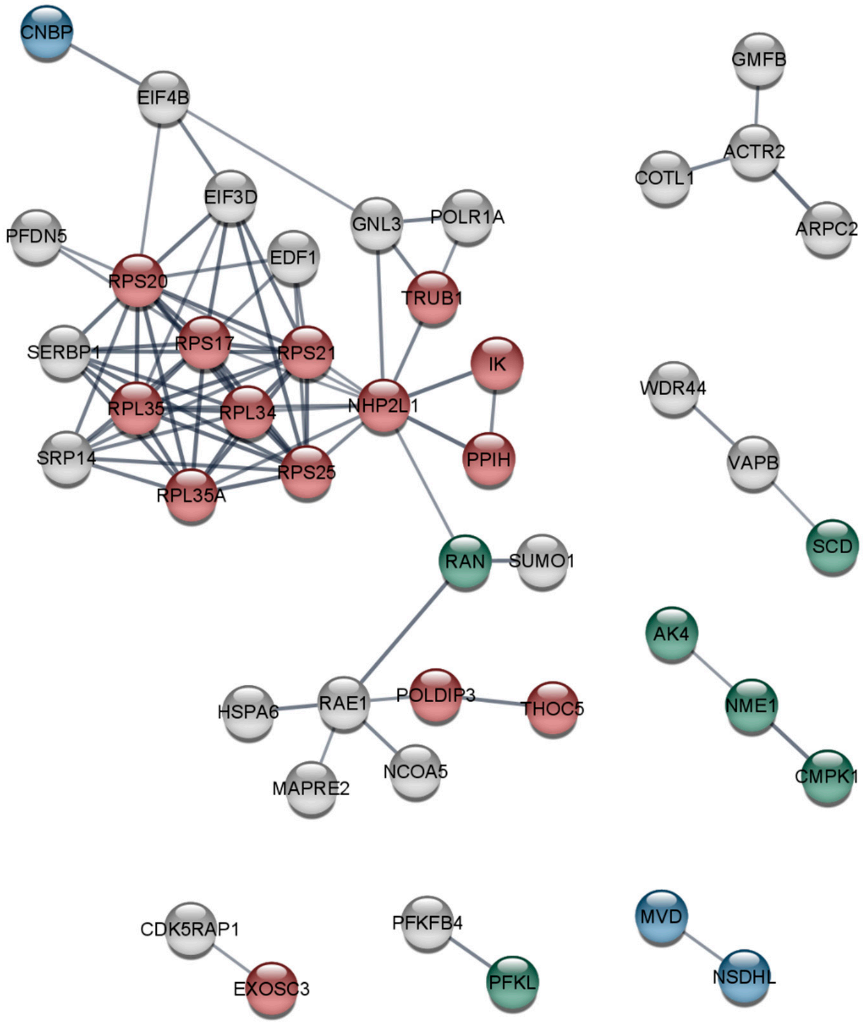

GO-term	strength	FDR
Cholesterol metabolic process	1.02	0.0115
Ribonucleotide metabolic process	0.77	0.0104
mRNA metabolic process	0.74	1.63e-05

Figure S7: String analysis of proteins exhibiting a ≥-1.5-fold change following OA500 treatment. The minimum required interaction score was set to 0.7 (high confidence) and disconnected nodes were excluded from the display. Nodes were coloured correspondingly to selected GO biological process search results. OA treatment resulted in a downregulation of ribosomal proteins indicating a reduction of cellular growth. OA500: 500 µM oleic acid for 24 h.

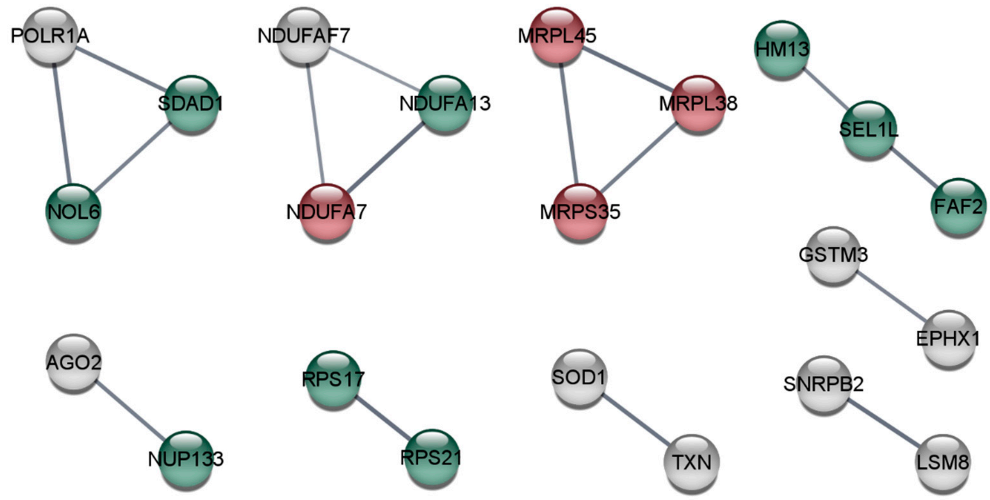

GO-term	strength	FDR
Mitochondrial gene expressio	1.17	0.0200
Intracellular protein transport	0.74	0.0021

Figure S8: String analysis of proteins exhibiting a ≥1.5-fold change comparing MA250 treatment to MO21. The minimum required interaction score was set to 0.7 (high confidence) and disconnected nodes were excluded from the display. Nodes were coloured correspondingly to selected GO biological process search results. Compared to the prevention treatment, MA250 presented with an upregulation of proteins associated with mitochondrial and cytoplasmatic protein synthesis. MA250: 250 µM myristic acid for 24 h. MO21: 250 µM myristic acid: 125 µM oleic acid for 24 h.

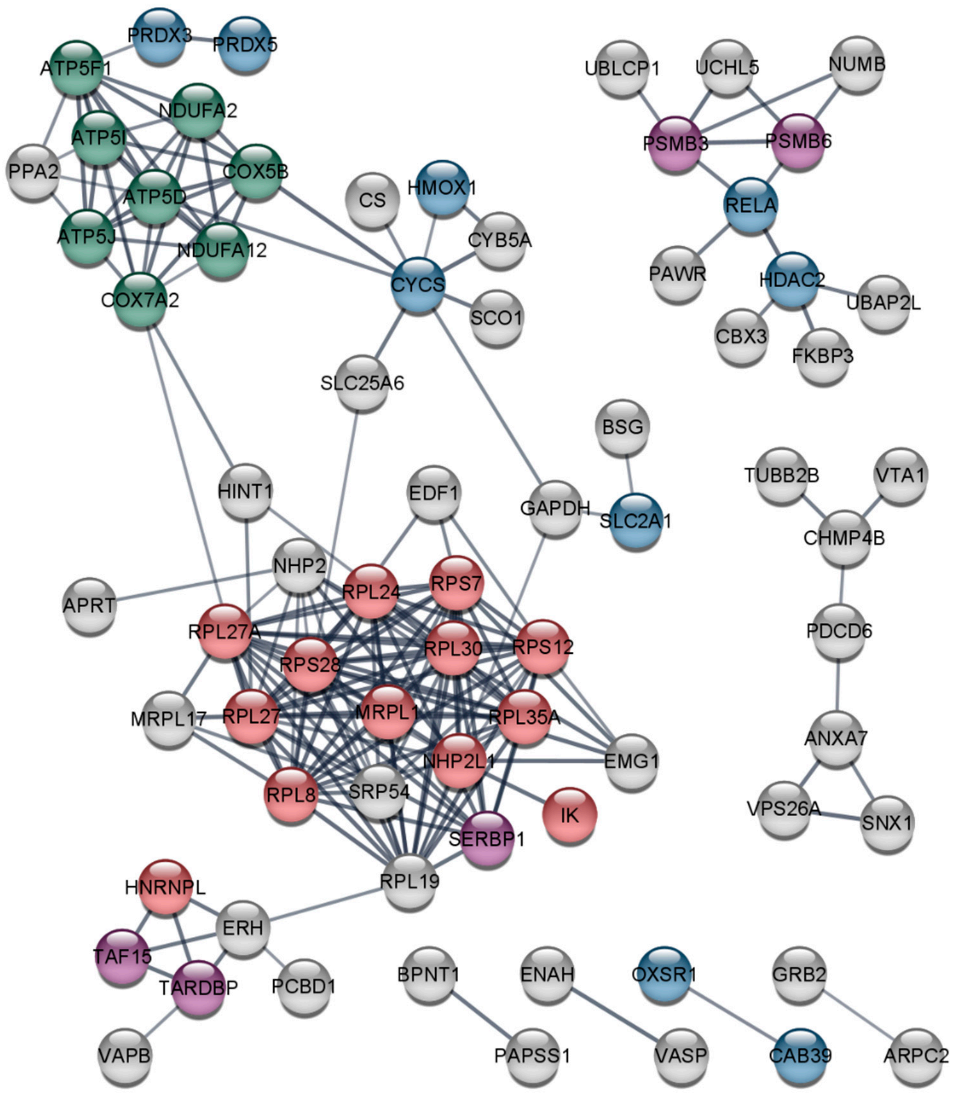

GO-term	strength	FDR
Oxidative phosphorylation	1.02	0.00017
Regulation of mrna catabolic process	0.73	0.0253
Cellular response to chemical stress	0.71	0.0034
mRNA metabolic process	0.61	8.66e-05

Figure S9: String analysis of proteins exhibiting a ≥-1.5-fold change comparing MA250 treatment to MO21. The minimum required interaction score was set to 0.7 (high confidence) and disconnected nodes were excluded from the display. Nodes were coloured correspondingly to selected GO biological process search results. In the prevention treatment proteins associated with oxidative phosphorylation and ribosomal proteins were found to be more abundant. The overabundance of ribosomal proteins corroborated the increase of proliferation rates back to control levels. MA250: 250 µM myristic acid for 24 h. MO21: 250 µM myristic acid: 125 µM oleic acid for 24 h.

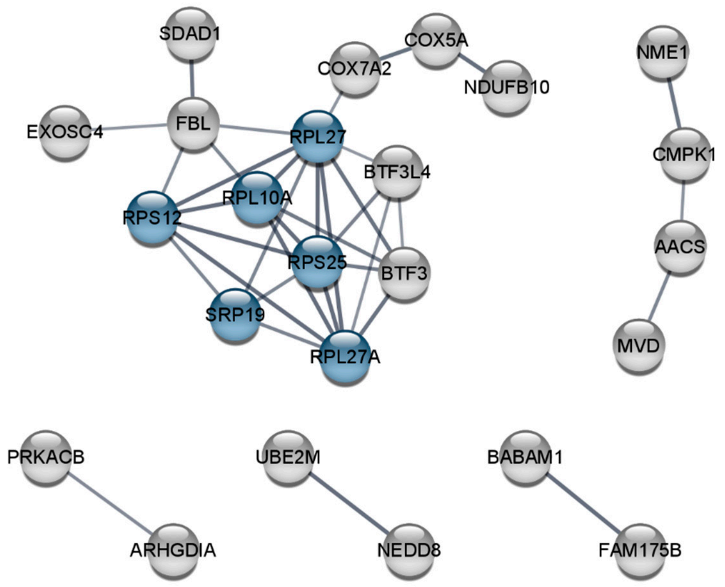

GO-term	strength	FDR
Protein targeting to er	1.3	0.0015

Figure S10: String analysis of proteins exhibiting a ≥1.5-fold change comparing PA250 treatment to PO21. The minimum required interaction score was set to 0.7 (high confidence) and disconnected nodes were excluded from the display. Nodes were coloured correspondingly to selected GO biological process search results. Compared to the prevention treatment, a subset of ribosomal proteins was found to be significantly enriched following PA treatment. PA250: 250 µM palmitic acid for 24 h. PO21: 250 µM palmitic acid: 125 µM oleic acid for 24 h.

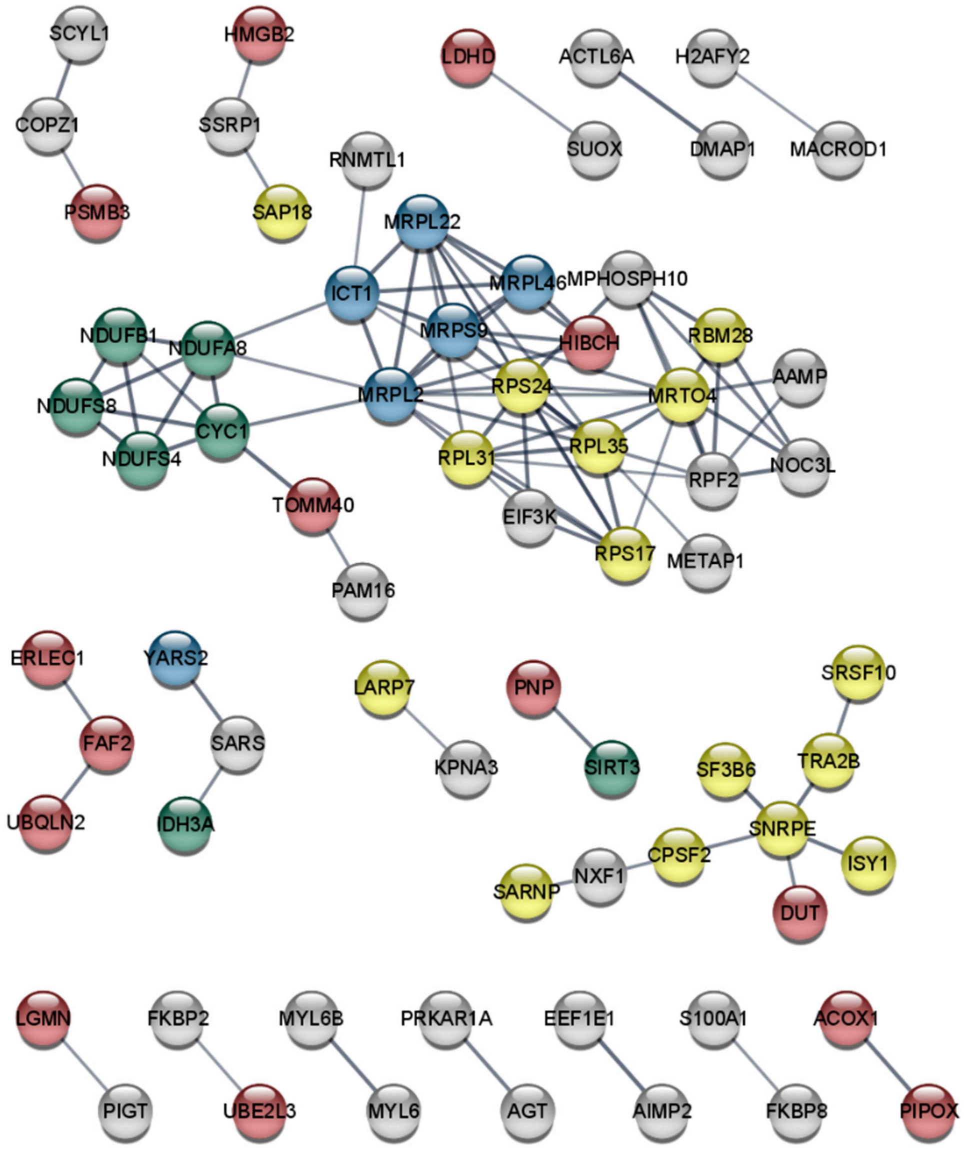

GO-term	strength	FDR
Mitochondrial translation	0.92	0.0312
Cellular respiration	0.9	0.0049
mRNA metabolic process	0.62	0.00029
Cellular catabolic process	0.47	1.52e-05

Figure S11: String analysis of proteins exhibiting a ≥-1.5-fold change comparing PA250 treatment to PO21. The minimum required interaction score was set to 0.7 (high confidence) and disconnected nodes were excluded from the display. Nodes were coloured correspondingly to selected GO biological process search results. Proteins associated with mitochondrial protein synthesis and oxidative phosphorylation, as well as a subset of ribosomal proteins was found to be significantly enriched in the prevention treatment. PA250: 250 µM palmitic acid for 24 h. PO21: 250 µM palmitic acid: 125 µM oleic acid for 24 h.
